# Cardiorespiratory Fitness, Metabolic Risk, and Inflammation in Children

**DOI:** 10.1155/2012/270515

**Published:** 2012-01-24

**Authors:** Antonios D. Christodoulos, Helen T. Douda, Savvas P. Tokmakidis

**Affiliations:** Department of Physical Education and Sport Science (T.E.F.A.A.), Democritus University of Thrace, 69100 Komotini, Greece

## Abstract

The aim of this study was to investigate the independent associations among cardiorespiratory fitness, metabolic syndrome (MetS), and C-reactive protein (CRP) in children. The sample consisted of 112 children (11.4  ±  0.4 years). Data was obtained for children's anthropometry, cardiorespiratory fitness, MetS components, and CRP levels. MetS was defined using criteria analogous to the Adult Treatment Panel III definition. A MetS risk score was also computed. Prevalence of the MetS was 5.4%, without gender differences. Subjects with low fitness showed significantly higher MetS risk (*P* < 0.001) and CRP (*P* < 0.007), compared to the high-fitness pupils. However, differences in MetS risk, and CRP between fitness groups decreased when adjusted for waist circumference. These data indicate that the mechanisms linking cardiorespiratory fitness, MetS risk and inflammation in children are extensively affected by obesity. Intervention strategies aiming at reducing obesity and improving cardiorespiratory fitness in childhood might contribute to the prevention of the MetS in adulthood.

## 1. Introduction

The prevalence and severity of obesity is increasing dramatically among children and adolescents in many parts of the world, whereas prevalence rates are estimated to increase in the next decades [1]. In children, excess body fat appears to be strongly associated with the clustering of risk factors, such as hyperglycemia, dyslipidemia, and hypertension, which play a key role in the pathogenesis of the metabolic syndrome (MetS) [2].

Obesity and the MetS risk in children have been recently associated with systemic inflammatory markers, in particular C-reactive protein (CRP) [3, 4], implying that low-grade inflammation can already exist in childhood and may be a potential link between the obesity and the MetS. Among behavioral variables, cardiorespiratory fitness has a protective role in MetS and inflammatory factors; however, it is not entirely clear if the interrelations among cardiorespiratory fitness, MetS risk, and inflammation in children are independent or partly due to the mediating effect of obesity, since the existing data are limited and equivocal [5, 6].

Recent evidence indicates that the prevalence rates of childhood obesity in Greece remain high [1, 7] and often coexist with low cardiorespiratory fitness [8] and an unfavorable cardiometabolic risk profile [9]. For the Greek pediatric population these data suggest an increased cardiovascular morbidity in adulthood, given that high-risk children and adolescents are likely to become high-risk adults [10]. Although the relationship among obesity and dyslipidemia in Greek children has been thoroughly investigated [9, 11], there is a paucity of data regarding the clustering of metabolic risk factors, inflammation, and their relationship with cardiorespiratory fitness. The present study was undertaken in an attempt to investigate the prevalence of the MetS and examine the associations among cardiorespiratory fitness, MetS risk, and CRP in 11-year-old children. 

## 2. Materials and Methods

### 2.1. Participants and Procedures

This study sample consisted of 112 pupils (54 females, 58 males, mean age 11.4 ± 0.4 years), registered in the sixth grade of 15 randomly selected primary schools in North Attica, Greece. Participants represented ~12% of the total 11-year-old population in the area. Subjects were healthy at the time of investigation and had no chronic or acute illnesses. Prior to the commencement of the study, permission from the school principal was obtained and parental consent was secured after full explanation of study objectives and data collection procedures. The children provided their verbal assent. The research ethics committee of Democritus University of Thrace approved the investigation and permission was granted by the Greek Ministry of Education. All procedures were in accordance with the Helsinki Declaration of 1975. Measures of anthropometry and fitness were conducted in the school environment (during PE classes) by the same experienced investigator (the principal author), using the same order of testing procedures and under similar conditions. Blood samples and blood pressure data were obtained at the same clinical biochemistry accredited laboratory by a medical practitioner. 

## 3. Data Collection

### 3.1. Anthropometry

Age (accurate to 1 month) was recorded. Students were weighed on an electronic scale to the nearest 0.5 kg (Seca Beam Balance 710), lightly dressed and barefooted. Standing height was measured to the nearest 0.5 cm (Seca Stadiometer 208) with each subject's shoes off, feet together, and head in the Frankfort horizontal plane. Body mass index (BMI) was calculated as weight divided by height squared (kg/m^2^). BMI values were also converted to standard deviation scores (SDSs), whereas the IOTF's age- and gender-specific BMI cut-off points were used to calculate the prevalence rates for “overweight/obesity” [12]. Waist circumference, measured at the level of the umbilicus with a tape measure to the nearest 0.1 cm, was used as an indicator of central obesity.

### 3.2. Cardiorespiratory Fitness

Cardiorespiratory fitness was assessed by using the 20 m multistage shuttle run test [13], a standard field test included in the European fitness test battery [14]. In brief, 5–10 subjects performed a series of runs across a 20 m track, changing direction at the end of each run to coincide with an audio signal that was getting progressively faster. Subjects started running at a speed of 8.5 km/hr, and speed increased at various stages (0.5 km/hr every minute). Each stage was made up of several shuttle runs, and pupils were instructed to keep pace with the signals as long as possible. The number of laps fully completed were recorded for each pupil and subsequently used to estimate cardiorespiratory fitness as predicted maximal oxygen uptake (VO_2max_) in mL/kg/min. Based on gender- and age-specific cut-off values of VO_2max_ [15] participants were classified into two fitness groups.

### 3.3. Clinical and Biochemical Screening

Blood pressure (BP) was measured in the right arm, using a mercury sphygmomanometer with appropriate size cuffs and cuff bladders, after the subject had been sitting quietly for at least 5 min. The mean of two measurements at the first and the fifth Korotkoff phase was recorded for systolic and diastolic BP, respectively [16].

Blood samples were obtained from each subject early in the morning, following an overnight fast. Blood was centrifuged for plasma separation, and 1.5 mL aliquots were pipetted into plastic Eppendorf tubes and stored at −70°C until further analyses. Plasma glucose, triglycerides, and HDL cholesterol (HDL-C) were determined using chromatographic enzymatic method on an automated MB II analyzer (Dade Behring, Marburg, Germany). CRP was measured by high-sensitive latex-enhanced immunoassay on a BN II nephelometer from the same company. The lower limit of detection of this assay was 0.15 mg/L. 

### 3.4. Metabolic Syndrome

The MetS was defined as meeting ≥3 of the following age- and gender-specific criteria: waist circumference ≥90th percentile, glucose ≥100 mg/dL, triglycerides ≥100 mg/dL, HDL-C < 50 mg/dL, and systolic or diastolic BP ≥ 90th percentile [17, 18]. We further computed a MetS risk score [19] as follows: for each of the MetS components, an absolute *Z *score was computed. For HDL-C the *Z *score was multiplied by −1 to indicate higher metabolic risk with increasing value. The *Z *scores of systolic and diastolic BP were averaged and then added to the rest of the *Z *scores. This sum was then divided by 5 to compile the average MetS risk score. This risk score is statistically more sensitive and less error-prone by comparison to dichotomous approaches [20]. We also computed a nonobesity MetS risk score, omitting the *Z *score from waist circumference.

### 3.5. Statistical Analysis

The Kolmogorov-Smirnov test of normality was adopted to assess data distribution. Because of a nonnormal distribution, CRP values were logarithmically transformed prior to the analyses. Descriptive statistics and simple contingency table analyses were performed. Continuous variables are presented as mean values ± standard error of means, while qualitative variables are presented as absolute and relative frequencies. Gender differences in the examined variables were explored using unpaired *t*-test. Relationships among cardiorespiratory fitness, MetS risk score, and CRP levels were summarized with Pearson's correlations. The independent effect of low fitness level on anthropometric indices, metabolic characteristics, MetS risk score, and CRP was assessed using one-way ANCOVA. The level of significance was set at *P* < 0.05. Statistical analyses were performed with SPSS 13.0 software.

## 4. Results

Descriptive characteristics of the subjects by gender and for the entire sample are presented in Table 1. Anthropometric parameters are in line with recently published paediatric data [1, 7, 8], suggesting that the sample is representative of periadolescent children in Greece. Mean BMI values, mean plasma concentrations of the MetS components, and mean BP recordings were within acceptable ranges and did not differ between genders. SDS-BMI was significantly higher in males compared to females (*t*_(110)_ = 2.52, *P* = 0.013).

Among all participants, 34.8% had one component, 12.5% had two components, 2.7% had three components, 1.8% had four components, and 0.9% had five components of the MetS. Hyperglycemia was the most common metabolic abnormality in both genders (27.7%), followed by hypertriglyceridemia (17.9%). Elevated BP was more frequent in females, but differences did not achieve statistical significance (*χ* ^2  ^ = 3.43, *P* = 0.064). The prevalence of the MetS was 5.4%, without statistically significant gender differences (*χ* ^2^ = 0.008, *P* = 0.928). 

Pearson's correlation analyses revealed that cardiorespiratory fitness was inversely associated with the MetS risk score (*r* = −0.386, *P* < 0.001), and CRP (*r* = −0.394, *P* < 0.001). MetS risk score and CRP were positively interrelated (*r* = 0.544, *P* < 0.0005). A high percentage of the study participants demonstrated cardiorespiratory fitness values considerably lower than the recommended fitness levels for metabolic health. In particular, ~72% of both genders did not achieve the acceptable fitness values for children of comparable age [15].

**Figure 1:**
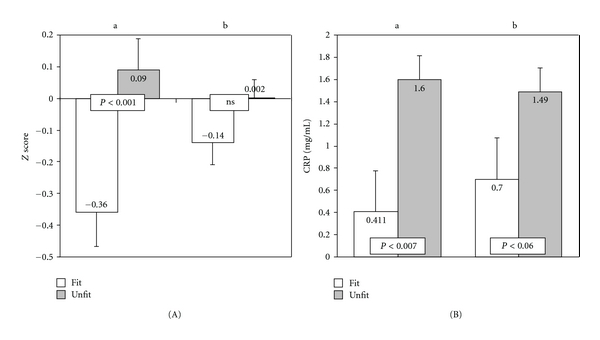
Differences in MetS risk score (A) and CRP values (B) between high- and low-fitness groups. (a) ANCOVA results after adjustment for age and gender. (b) ANCOVA results after further adjustment for waist circumference.

Children with lower fitness levels, compared to their peers with increased fitness levels, demonstrated significantly increased BMI (21.01 versus 17.91 kg/m^2^, *F*_(1,111)_ = 17.55, *P* < 0.001), SDS-BMI (1.07 versus 0.14, *F*_(1,111)_ = 13.43, *P* < 0.001) and waist circumference (72.89 versus 64.23 cm, *F*_(1,111)_ = 19.82, *P* < 0.001). In addition, pupils in the lower fitness group showed significantly higher MetS risk score (*F*_(1,93)_ = 13.03, *P* < 0.001) and CRP values (*F*_(1,93)_ = 7.67, *P* < 0.007), compared to the high-fitness group (Figure 1). Adjustment for central obesity attenuated the observed differences in the MetS risk score, which were no longer significant (Figure 1(A)). Differences in CRP values between fitness groups decreased also when adjusted for waist circumference (Figure 1(B)); however, they remained borderline significant (*F*_(1,93)_ = 3.49, *P* < 0.067). Analyses including BMI or SDS-BMI instead of waist circumference yielded similar results (data not shown).

## 5. Discussion

The MetS has a multifactorial aetiology, and the mechanisms underlying its onset are not completely understood. In the present study we investigated the prevalence of the MetS and examined the independent associations among cardiorespiratory fitness, MetS risk, and CRP in Greek children. Using a paediatric definition based closely on ATP III [17, 18], we found that the prevalence of MetS in our sample was 5.4%. This prevalence rate is similar to those reported in US and French adolescents (4–5.8%) [21–23] but lower than the prevalence reported in Iranian paediatric populations (14.1%) [24]. These data indicate that the MetS is a common health problem among different paediatric populations, underlining the need for effective policies and appropriate intervention strategies at global, national, and regional level.

We further found that the protective effect of cardiorespiratory fitness on the MetS risk score was explained by the confounding effect from central obesity. Our results are in agreement with previous findings [6], showing that cardiorespiratory fitness is not independently associated with markers of the MetS. However, recent data from the European Youth Heart Study (EYHS) [19, 25, 26] and the National Health and Nutrition Examination Survey (NHANES) 1999–2002 [27] revealed that cardiorespiratory fitness is a significant and independent predictor of the MetS in children, whereas the benefits of higher cardiorespiratory fitness seem to extend also to children at lower metabolic risk [28]. Differences in body composition and fitness levels are known to affect the metabolic profile in children and could explain at least part of these discrepancies among studies.

Compared to their North European and North American age-related peers, Greek children exhibit higher rates of overweight/obesity and worse cardiorespiratory fitness performance [8, 19, 27, 29–31]. These findings suggest that children in the present study did not achieve the required threshold in cardiorespiratory fitness, in order to profit from its beneficial effects with respect to the risk of MetS in the face of obesity. Indeed, Nassis et al. [32] have shown that exercise-related cardiorespiratory fitness enhancement in overweight girls can lead to marked improvements in insulin sensitivity, without changes in body composition. In addition, even if central obesity is an intermediate link between low fitness and MetS risk, fitness will still be the principal cause, even if its association with MetS risk disappears after adjustment for an intermediate variable in the causal chain (i.e., central obesity). 

In line with a previous report in children and young adults [5], we found that the association between cardiorespiratory fitness and CRP was largely explained by their interrelation to obesity. Thomas et al. [33] reported no relationship between CRP and cardiorespiratory fitness in 12-13-years-old schoolchildren. In contrast, results from the Ten Towns Children's Study [34] and more recent data from South African children [35] reported strong correlations between fitness and CRP. An explanation for this discrepancy may be the fact that in the Ten Towns Children's Study a crude estimate of physical fitness (i.e., resting heart rate) was used, whilst in the latter study [35] the investigators did not take into account the confounding effect of obesity. More studies are needed to improve our understanding of the interrelations among cardiorespiratory fitness, CRP, and obesity in children.

Our findings indicate that the mechanisms linking cardiorespiratory fitness, MetS risk, and CRP are extensively affected by obesity. Further, the favourable effect of cardiorespiratory fitness on MetS risk may not become evident when fitness levels underlie a critical threshold. Given that high levels of cardiorespiratory fitness improve the metabolic state of obese children [32], attenuate the proinflammatory milieu associated with obesity [36], and offer protection against the development of the insulin resistance syndrome from childhood into adolescence and young adulthood [37–39], children should be encouraged to achieve the recently recommended physical activity levels (90 min/day of at least moderate intensity), in order to prevent clustering of cardiovascular disease risk factors and improve their metabolic and overall health profile [28]. 

The interpretation of the present findings may be affected by several potential limitations. A root cause in the development of the MetS is insulin resistance, which was not assessed in the present study. However, epidemiologic studies indicate that a substantial proportion of subjects with the MetS do not have evidence of insulin resistance and the correlation between insulin resistance and individual components of the MetS is weak to moderate [40]. In addition, cardiorespiratory fitness was indirectly assessed. However, it is unlikely that our findings are invalidated by the use of the 20 m multistage shuttle run test since it has been extensively used in the literature and is recommended for paediatric studies as reliable, valid, and cost-effective [8, 13, 14, 30, 31]. Further, a strict random sampling of all eligible pupils was virtually impossible, given that the study was dependent on the principals' disposal and the willingness of individual children and their parents to participate. The prevalence of overweight/obesity, however, was similar to that reported for school-aged children from other Greek regions [7–9, 11]. Finally, the sample size and the age range of our subjects limit the generalization of the findings.

Within the above-mentioned limitations, it is concluded that the metabolic consequences of excessive body fat and low fitness are detectable early in life. Taking into consideration the growing epidemic of childhood obesity, the public health authorities should seriously consider the design and early implementation of effective prevention strategies. Health education programs aiming at the prevention of weight gain and the improvement of cardiorespiratory fitness in childhood could anticipate the metabolic abnormalities predisposed to or associated with MetS risk and inflammation and thus contribute to the prevention of the MetS and its complications later in life.

## Figures and Tables

**Table 1:** Subject characteristics and prevalence rates of metabolic risk factors and the MetS.

	Females (*n* = 54)	Males (*n* = 58)	*P* value	Overall (*n* = 112)
Anthropometric and fitness characteristics				
Age (years)	11.28 ± 0.04	11.41 ± 0.05	0.06	11.35 ± 0.04
Height (cm)	150.86 ± 0.85	150.78 ± 0.87	0.94	150.82 ± 0.61
Weight (kg)	44.98 ± 1.43	47.05 ± 1. 45	0.31	46.05 ± 1.02
BMI (kg/m^2^)	19.65 ± 0.53	20.54 ± 0.51	0.23	20.11 ± 0.37
SDS-BMI	0.49 ± 0.18	1.08 ± 0.15	0.01	0.79 ± 0.12
Normal, *n* (%)	34 (63)	33 (56.9)		67 (59.8)
Overweight/obese, *n* (%)	20 (37)	25 (43.1)	0.513	45 (40.2)
VO_2max⁡_ (mL/kg/min)	29.86 ± 0.72	31.26 ± 0.79	0.15	30.58 ± 0.54

*MetS components and CRP concentration*				
Waist circumference (cm)	68.83 ± 1.29	71.97 ± 1.47	0.12	70.47 ± 0.99
≥90th percentile, *n* (%)	6 (11.1)	8 (13.8)	0.67	14 (12.5)
Fasting glucose (mg/dL)	93.58 ± 1.33	95.36 ± 1.26	0.30	94.51 ± 0.92
≥100 mg/dL, *n* (%)	13 (24.1)	18 (31.0)	0.41	31 (27.7)
HDL-C (mg/dL)	63.54 ± 1.73	63.68 ± 1.66	0.97	63.62 ± 1.19
<50 mg/dL, *n* (%)	9 (16.7)	6 (10.3)	0.33	15 (13.4)
Triglycerides (mg/dL)	73.74 ± 6.16	70.15 ± 5.05	0.60	71.88 ± 3.93
≥110 mg/dL, *n* (%)	10 (18.5)	10 (17.2)	0.86	20 (17.9)
Systolic BP (mmHg)	100.67 ± 1.08	102.68 ± 1.04	0.19	101.71 ± 0.75
Diastolic BP (mmHg)	75.96 ± 0.78	76.16 ± 0.70	0.85	76.06 ± 0.52
≥90th percentile, *n* (%)	7 (13)	2 (3.5)	0.06	9 (8)
MetS prevalence (≥3 risk factors), *n* (%)	3 (5.6)	3 (5.2)	0.93	6 (5.4)
MetS Z score including adiposity	−0.09 ± 0.09	0.04 ± 0.07	0.45	−0.02 ± 0.06
MetS Z score excluding adiposity	−0.08 ± 0.09	0.00 ± 0.06	0.78	−0.04 ± 0.05
CRP (mg/dL)	1.07 ± 0.22	1.47 ± 0.32	0.28	1.27 ± 0.19

Values are presented as means ± SEM or *n* (%). BMI: body mass index, BP: blood pressure, CRP: C-reactive protein, HDL-C: high-density lipoprotein cholesterol, MetS: metabolic syndrome, SDS-BMI: standard deviation scores for BMI, and VO_2max⁡_: predicted maximal oxygen uptake.
